# Supplementary material for: Flexible route planning and rapid structure learning by mice in complex environments
Source: bioRxiv. 2026 Jun 5:2026.06.02.729586. Preprint. [Version 1] doi: 10.64898/2026.06.02.729586 (PMC13251939; doi:10.64898/2026.06.02.729586)
Supplement: Supplement 1 [file NIHPP2026.06.02.729586v1-supplement-1.pdf]

bioRxiv preprint doi: https://doi.org/10.64898/2026.06.02.729586; this version posted June 5, 2026. The copyright holder for this preprint (which was not certified by peer review) is the author/funder, who has granted bioRxiv a license to display the preprint in perpetuity. It is made available under a CC-BY 4.0 International license.

Supplementary Figures

bioRxiv preprint doi: https://doi.org/10.64898/2026.06.02.729586; this version posted June 5, 2026. The copyright holder for this preprint (which was not certified by peer review) is the author/funder, who has granted bioRxiv a license to display the preprint in perpetuity. It is made available under a CC-BY 4.0 International license.

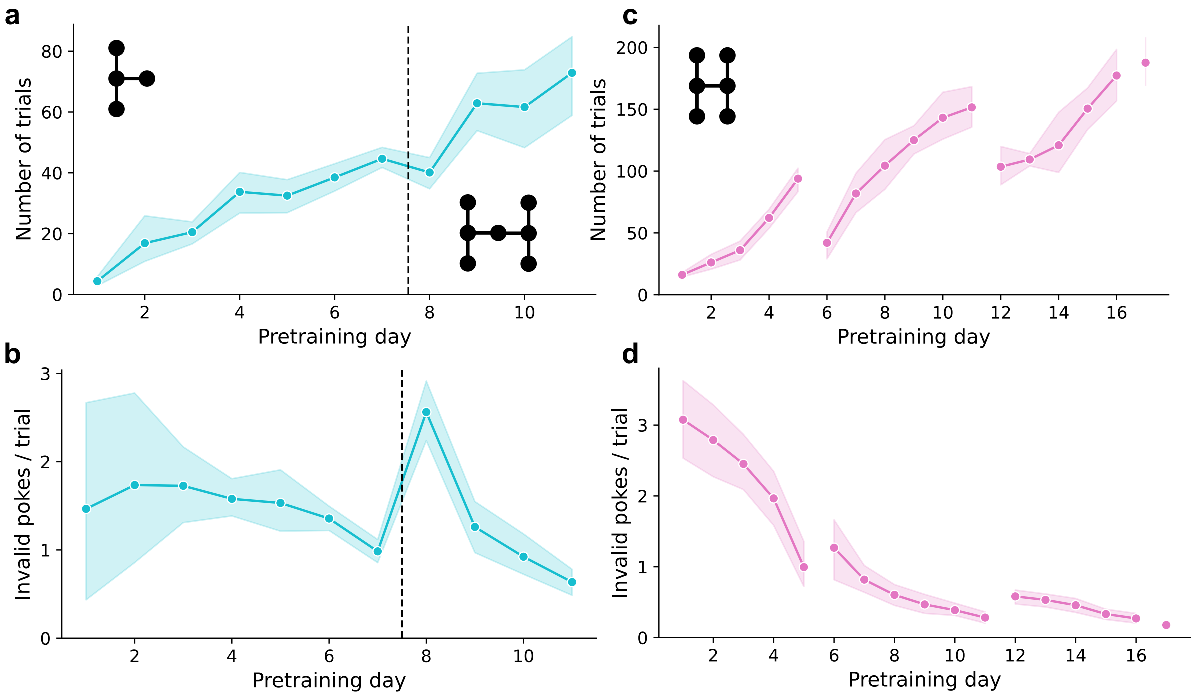

Figure S1: **Pretraining behaviour. a)** Number of trials per pretraining day in the pilot experiment. For the first 7 days of pretraining the maze configuration was a 4 node "T" shape, for the last 4 days of pretraining the maze configuration was a 7 node "H" (black inserts). **b)** Invalid pokes per trial - i.e. pokes in non-goal locations, across pretraining days in pilot experiment. **c)** Number of trials per pretraining day in the main experiment. For all days the maze configuration was a 6 node "H". **d)** Pretraining invalid pokes per trial in main experiment. Lines show the mean across subjects, shaded regions show the 95% confidence interval. Lines are disconnected across non-consecutive calendar days.

bioRxiv preprint doi: https://doi.org/10.64898/2026.06.02.729586; this version posted June 5, 2026. The copyright holder for this preprint (which was not certified by peer review) is the author/funder, who has granted bioRxiv a license to display the preprint in perpetuity. It is made available under a CC-BY 4.0 International license.

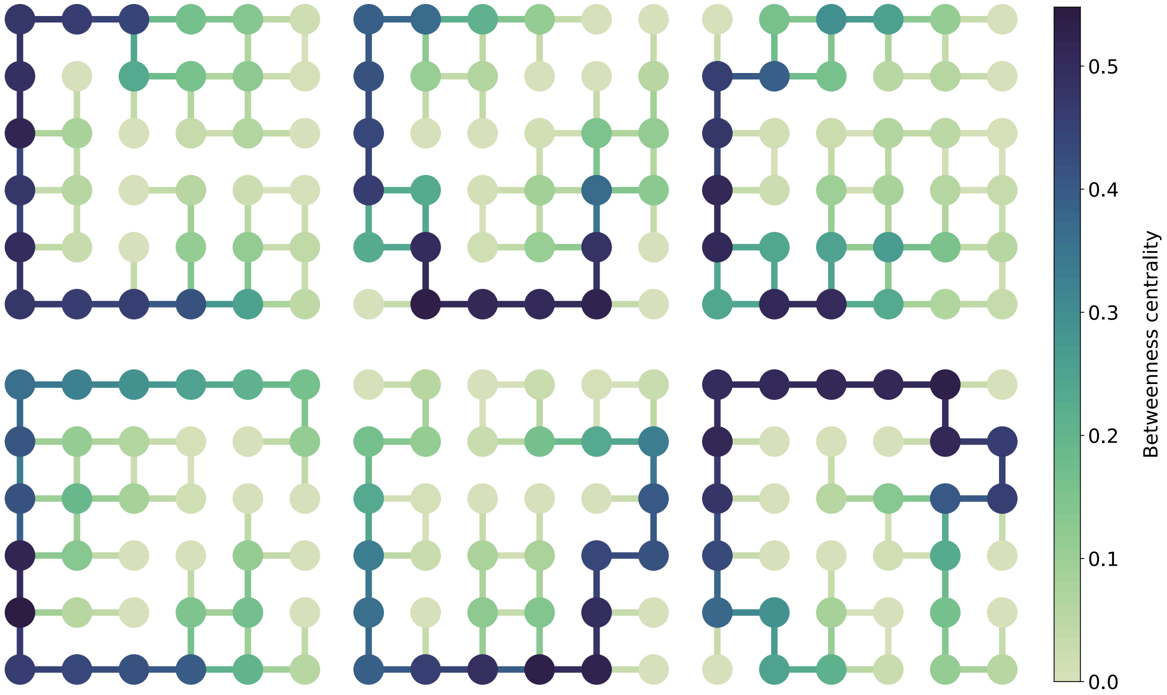

Figure S2: **Optimised mazes:** Top 6 candidate maze layouts generated by our maze search procedure when ranked by fraction of informative states in descending order. Note the prevalence of a prominent linear structure folded into the 6x6 grid.

bioRxiv preprint doi: https://doi.org/10.64898/2026.06.02.729586; this version posted June 5, 2026. The copyright holder for this preprint (which was not certified by peer review) is the author/funder, who has granted bioRxiv a license to display the preprint in perpetuity. It is made available under a CC-BY 4.0 International license.

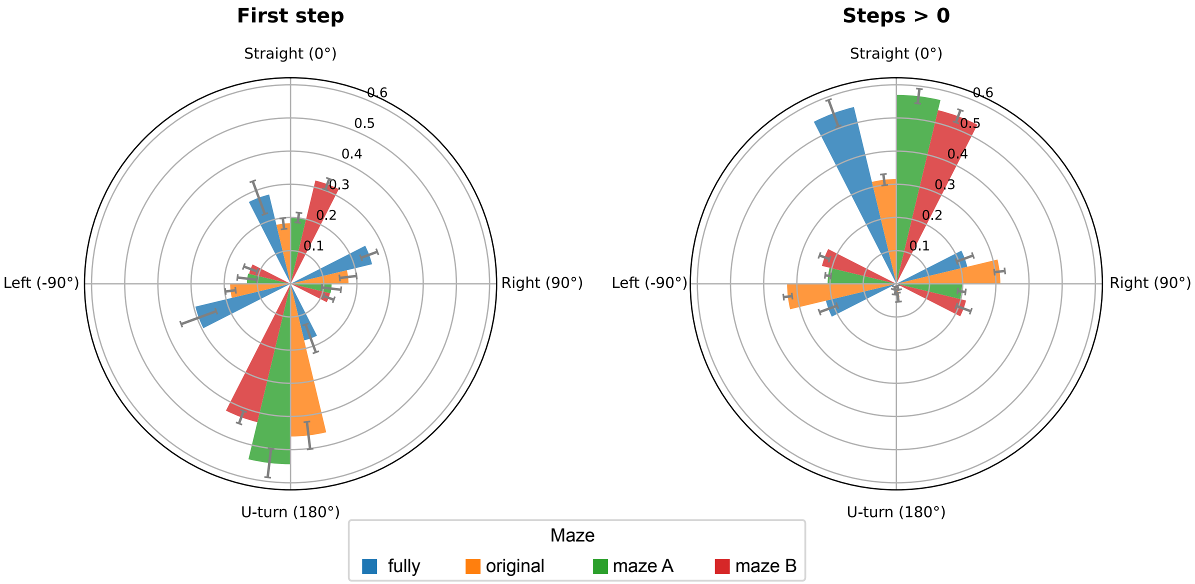

Figure S3: **Egocentric action bias:** Rate of taking the 4 possible egocentric actions; straight-on, left-turn, right-turn, u-turn, across the 4 mazes of experiment 2 (indicated by colour). Shown separately for the first step following goal cue onset (left panel) and for all subsequent steps during navigation to goal (right panel). The analysis was restricted to locations with more than one available action, excluding dead ends where the only possible movement is back along the previous path.

bioRxiv preprint doi: https://doi.org/10.64898/2026.06.02.729586; this version posted June 5, 2026. The copyright holder for this preprint (which was not certified by peer review) is the author/funder, who has granted bioRxiv a license to display the preprint in perpetuity. It is made available under a CC-BY 4.0 International license.

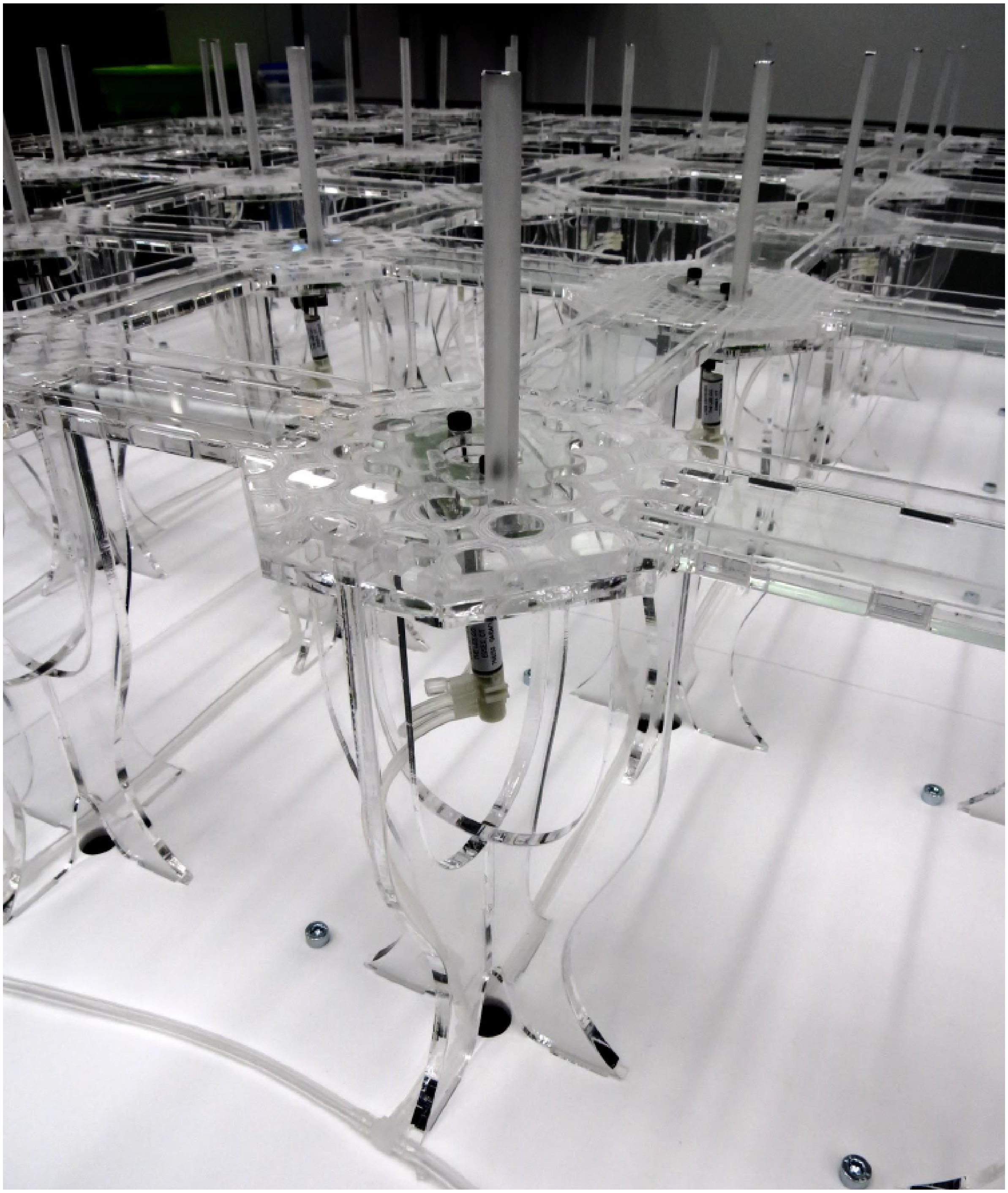

Figure S4: **Tower and walkway construction** Photo showing close-up of a tower and connected walkways. The solenoid used to control reward delivery can be seen under the tower. An acrylic rod illuminated by the goal-cue LED sticks up vertically from the tower top.

bioRxiv preprint doi: https://doi.org/10.64898/2026.06.02.729586; this version posted June 5, 2026. The copyright holder for this preprint (which was not certified by peer review) is the author/funder, who has granted bioRxiv a license to display the preprint in perpetuity. It is made available under a CC-BY 4.0 International license.

Supplementary Tables

Table S1: Model comparison summary. Bullets mark predictors included in each candidate model. '_x' suffix indicates interaction with experience on maze, Freq. = estimated population model frequency, PXP = protected exceedance probability.

Model	Anti-backward	Anti-backward_x	Vector	Vector_x	Structure	Structure_x	Freq.	PXP
1	●		●		●		0.000	0.002
2	●		●	●	●		0.000	0.002
3	●	●	●	●	●		0.000	0.002
4	●		●		●	●	0.000	0.002
5	●	●	●		●	●	0.000	0.002
6	●		●	●	●	●	1.000	0.989
7	●	●	●	●	●	●	0.000	0.002
